# YIMBY—Yes, In My BackYard!—The successful transition to a local online ecology field course

**DOI:** 10.1002/ece3.6881

**Published:** 2020-10-07

**Authors:** Laura McKinnon

**Affiliations:** ^1^ Department of Multidisciplinary Studies and Graduate Program in Biology York University Toronto Ontario Canada

**Keywords:** experiential education, field course, online, urban ecology

## Abstract

Field biology courses provide the ultimate experiential education as students discover the links between theory and practice in ecology and evolution directly in nature. During the spring and summer of 2020, the COVID‐19 pandemic led to the cancelation of face‐to‐face classes in almost every university in Canada. Whereas traditional university courses were mostly transferred online, the online transition for field biology courses was not so common. Here, I provide an account of a successful transition from traditional field biology course to an online “backyard biology” field course with a small class size of 10 students. While the online field course may not provide the same level of interpersonal benefits of the traditional field course experience, the model outlined here demonstrates that an online field course that incorporates direct experience with the natural environment is possible and should no longer be considered an oxymoron.

## Graphical abstract

Field biology courses provide the ultimate experiential education. Here I provide an account of a successful transition from traditional field biology course to an online ‘backyard biology’ field course. While the online field course may not provide the same level of interpersonal benefits of the traditional field course experience, the model outlined here demonstrates that an online field course that incorporates direct experience with the natural environment is possible and should no long be considered an oxymoron.

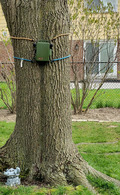

## 1 INTRODUCTION

Almost every field ecologist will attest to the benefits, or some may even say, the absolute necessity, of a good old‐fashioned field course to ensure a well‐rounded undergraduate degree in biology. Though it may come in a variety of forms, at its simplest, the field course is best defined by Fleischner et al. (2017) as any “educational experience that incorporates direct experience with the natural environment.” Field courses provide the ultimate experiential education in ecology. After years of sitting through hours of lectures and well‐planned laboratory exercises, the field course is often the first opportunity for students to learn a variety of techniques in ecological research while in the field—the classroom is literally outdoors and hands‐on. Field courses increase academic performance in biology (Easton & Gilburn, 2012) and expose students to new ways of learning in a novel setting (Fleishner et al., 2017). The experience of a field course can be so transformative that it is not uncommon for students to change directions late in their degree to pursue passions in field ecology that they never knew existed.

In 2016, I developed a third‐year undergraduate field course entitled “Ecological Monitoring in an Urban Environment” and have been delivering it successfully at York University, Glendon Campus, Toronto, ON, Canada, each year since. The course is usually offered as part of the Ontario Universities Program in Field Biology (OUPFB: http://www.oupfb.ca/); therefore, it has a standard OUPFB format of 12 days of field activities with a comprehensive report due at the end of the course. Unlike many other OUPFB courses that are held in exotic natural locations, the course is held in the heart of downtown Toronto ON at York University's Glendon Campus. Fortunately, Glendon Campus is located in the secluded Don River which is part of Toronto's extensive ravine system, making our field site essentially a large island of greenspace in the center of Canada's largest city. The field course combines short interactive lecture sessions (<2 hr) with daily field activities on campus and usually 2 or 3 excursions to other natural areas in the Greater Toronto Area. Like other field courses, the days are long, and the majority of time is spent outdoors. The daily field activities cover a broad range of ecological sampling topics. On ornithology, we generally include three mornings of passerine banding using mist nets (starting at sunrise for 6 hr), one morning searching for color banded American Robins (*Turdus migratorius*), and one morning searching for Killdeer (*Charadrius vociferus*) nests in a nearby park. To gain field experience in entomology, students setup malaise and pitfall traps, sample these traps for five days, and spend at least one‐half day identifying their samples to family. They also collect data on habitat structure at their sampling sites and explore the diversity of mammals using wildlife traps for four days. To gain field skills in herpetology, we spend one evening conducting amphibian surveys at a local wetland and one day catching turtles as part of a capture‐mark‐recapture program with other York University researchers and the Toronto Region Conservation Authority. In 2019, while donning waders to check turtle traps, students actively participated in the find of a rare Eastern Musk Turtle (*Sternotherus odoratus*), a species that had not been seen in the area since 1969 (Dupuis‐Désormeaux et al., 2019).

During the second week of the course, the students apply the techniques learned to a group research project, which encompasses a written proposal, a proposal defense seminar, and a final scientific paper (see Appendix S1 for project examples). How could all these field experiences ever be replicated in an online environment? It is hard to imagine, but knowing that some students would not be able to graduate without this last mandatory field course, and with the risk of having to abandon field course teaching for an additional lecture‐based course in the fall, I quickly set about to make this work.

## 2 DESIGNING THE ONLINE FIELD EXPERIENCE

Many would argue that an online field course is an oxymoron. A few short months ago, I would have agreed. However, in my last‐minute rush to put this course together, I kept Fleischner et al.’s (2017) simple definition in mind “educational experience that incorporates direct experience with the natural environment.” Under normal circumstances, this would still be a challenge. Field exercises that required the presence of skilled and permitted instructors such as bird banding, nest searching, and turtle trapping would have to go. This is unfortunate as those activities were always student favorites (and mine). With the COVID‐19 pandemic, however, the constraints multiplied. To adhere to the new sanitary guidelines, including social distancing, I could not ask students to leave their homes to go birding or even visit a wetland to survey calling frogs. There was really only one solution. All field activities had to take place in the students’ backyards.

Luckily, the course the students needed to graduate was “ecological monitoring in an urban environment” so any size of private greenspace, regardless the level of urbanization, would work. Once I confirmed that all students had access to some semblance of private greenspace (backyard or front yard) where they would not have contact with anyone outside their household, I started to brainstorm appropriate backyard field activities. Given that students would be doing all the work in their backyard, I wanted them to be well equipped. Good ecological monitoring equipment can be expensive but can really augment the field experience for students. Thanks to generous internal grants that encourage experiential education, in my field courses I am able to provide students with everything they need to conduct research like a real field biologist. They each get binoculars, field notebooks, field guides, GPS, dissecting scope etc., any of the equipment the fieldwork would require in the real world. For the online field course, I still wanted to give this experience, so another condition of the course was that students were required to pickup and return a box of equipment, following social distancing guidelines (i.e., no public transit). Due to equipment constraints, the usual enrollment maximum of 20 was reduced to 10 students. Eight of ten students were able to meet these two conditions. The two students that could not meet the conditions were international students, however, I could easily foresee situations in which many more students, even if living locally, could not (limited home computer or internet access, living in an urban high‐rise, no access to family vehicle etc.). Admittedly, these types of conditions can certainly hinder the promotion of equity, diversity, and inclusion in field courses and need to be considered should the course be delivered in the future. That said, the online field course was adapted rapidly in the face of the pandemic, and future online offerings could be much more flexible. Indeed, outside of the specific constraints of the pandemic, there is potential for the “backyard biology” model presented here to increase equity, diversity, and inclusion as it provides a valuable alternative field course experience for students with financial and/or mobility/health issues that prohibit them from taking a traditional field biology course. I have already started a modified version greatly reducing these constraints for international students. This will be yet another interesting experience.

Once the shock of the course constraints subsided, planning the content of what was essentially a “backyard biology” course was not so difficult. Below I provide details of the activities conducted during the course.

## 3 GENERAL FIELD OBSERVATIONS

Students were provided a standard SitePro 350‐T case bound field book (Universal Field Supplies, Toronto, ON). One of our first videoconference sessions was dedicated to how to take efficient field notes. Students were required to outline all daily field activities in the field book as well as daily local weather conditions at the beginning of all exercises. Students documented presence of precipitation, percent cloud cover, wind speed based on the Beaufort wind scale and wind direction (TRCA, 2011). At the end of the course, field books were scanned and emailed to me for evaluation. All protocols followed during the course were posted on York's Learning Management System.

## 4 URBAN ORNITHOLOGY

**Figure 1:**
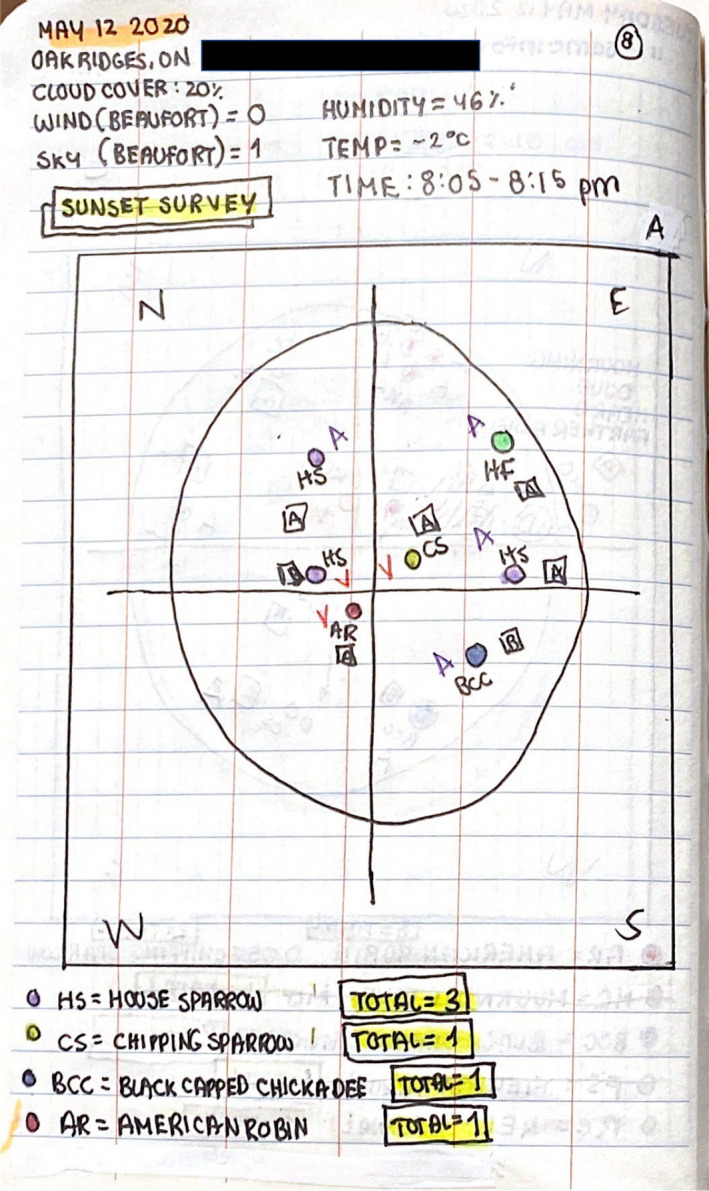
An example of the data collected during a sunrise point count from one students’ field notebook

**Figure 2:**
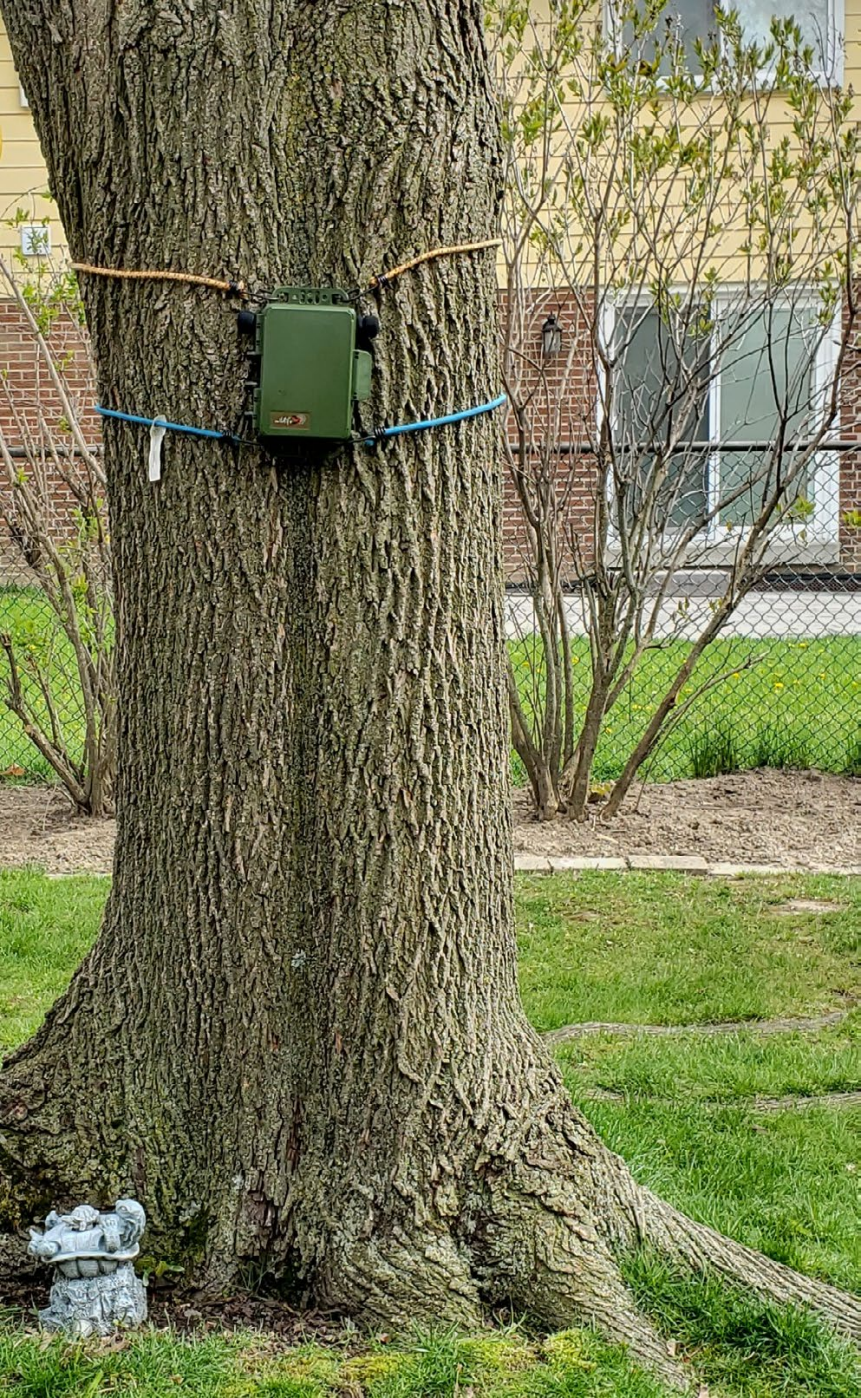
An example of the bird bioacoustics recorder deployment in one students’ backyard. Photograph credit: S. Nichols

As an avian ecologist, my field courses always have a large avian component (bird banding, nest searching, etc.) and I wanted to maintain this. Instead of bird banding, students focussed on learning to identify birds by sight and sound and monitoring avian diversity in their yards using point counts and bioacoustics monitors. Students were provided binoculars, a Sibley's Guide to the Birds of Eastern North America (Sibley, 2011), point count protocols (TRCA, 2011), and a SongMeter SM4 bioacoustic monitor (Maynard, MA, USA). Though I did post links to the many available instructional videos online, I tried to interact as much as possible with the students via videoconferencing. For example, instead of directing the students to the manual for their binoculars, I made everyone bring their binoculars to the screen, and I walked them through calibration of the binoculars step by step. In the same manner, I explained to them how best to find birds using binoculars, shared my screen to show them the most common urban birds they were likely to find, and provided tips on how to identify unknown birds by sight and sound. I also showed them how to access online applications (Merlin—https://merlin.allaboutbirds.org/, iNaturalist—https://www.inaturalist.org/) and websites (www.allaboutbirds.org) that are frequently used by professional ornithologists in the field so that students had numerous tools on hand for identification. For the first four days of the course, students completed 3 points counts (separated by 10‐min intervals) each morning at sunrise, and again at sunset (Figure 1). In short morning videoconference meetings, we would recap the new sightings or songs heard across the group. Sometimes students would share their photographs or videos of a new bird or a new bird song that they could not identify and we would share screens with each other as we searched online to help identify their new species. The students identified up to 28 bird species, of which up to 60% were new to students. On the second day, students deployed bioacoustics recorders in their yards, set to record for 2 hr centered on sunrise and sunset everyday (Figure 2). Two online analysis workshops were held, the first an introduction to Studio R and basic statistical tests (*t* test, linear regression, nonparametric alternatives), and the second an introduction to bioacoustics analysis. In the latter, students extracted wav files using Kaleidoscope 5.1.9i (Wildlife Acoustics Inc) and identified bird songs using two R packages, monitoR (Hafner & Katz, 2018) and tuneR (Ligges et al., 2018). Again, despite having posted the sample code with instructions online, students were required to download the necessary programs and use them live together. I used videoconferencing and a shared screen to walk everyone through the sample analyses step by step to make sure that each student would be able to run these analyses independently using their own data later in the course. As usual, there were glitches that arose, but when necessary, the students could share their own screen with the group in order to figure out the issue together. This was a very effective teamwork exercise for the students. Switching between applications (i.e., from Studio R, to Google Chrome, to KaleidoScope) while sharing screens was not always smooth, but students quickly got used to me asking repeatedly “are you with me now?” Overall, despite not catching birds in the hand, these activities certainly provided students with a set of advanced skills in ornithological research.

## 5 URBAN ENTOMOLOGY

**Figure 3:**
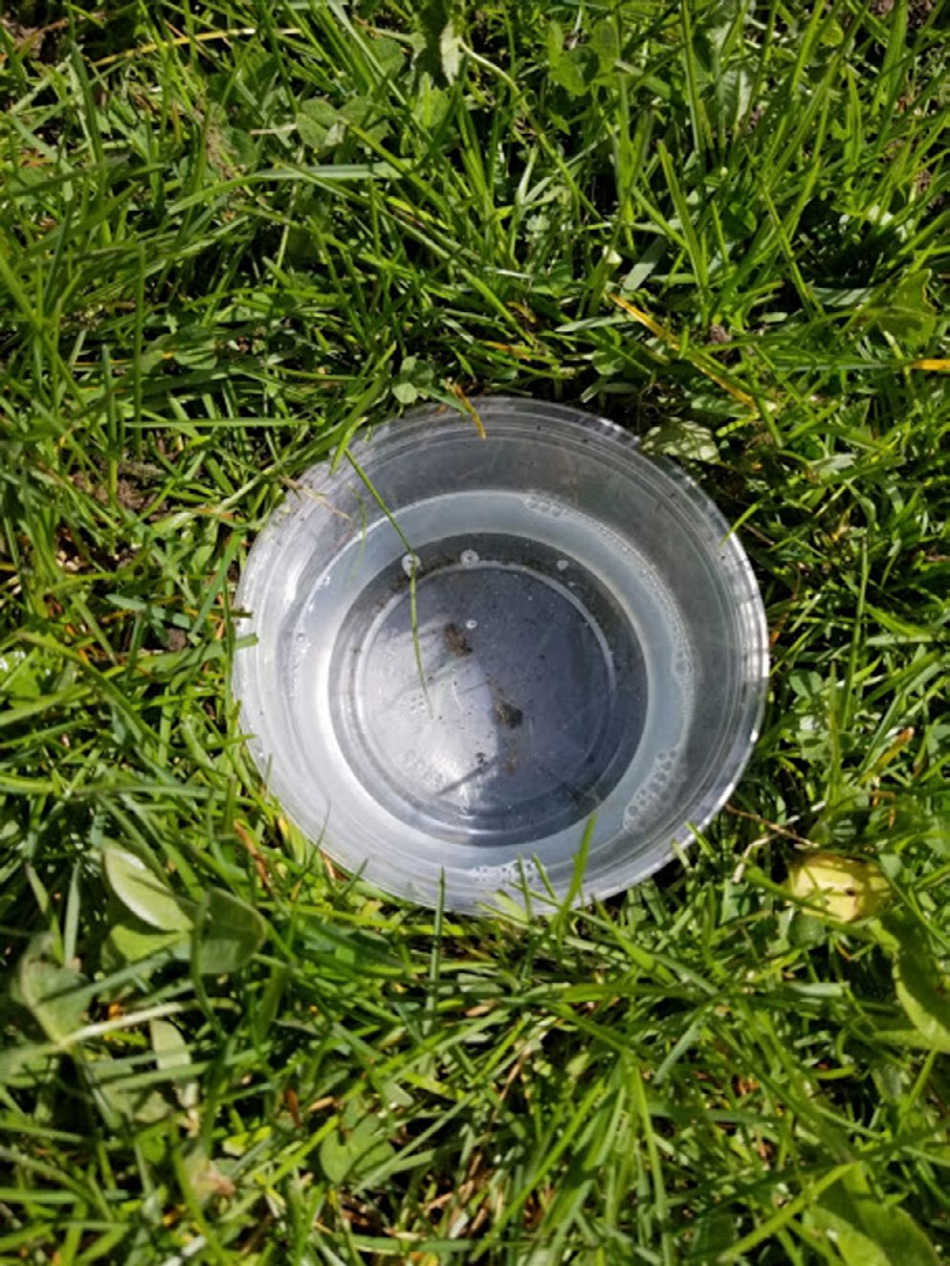
An example pitfall trap setup. Photograph credit: A. Malik

**Figure 4:**
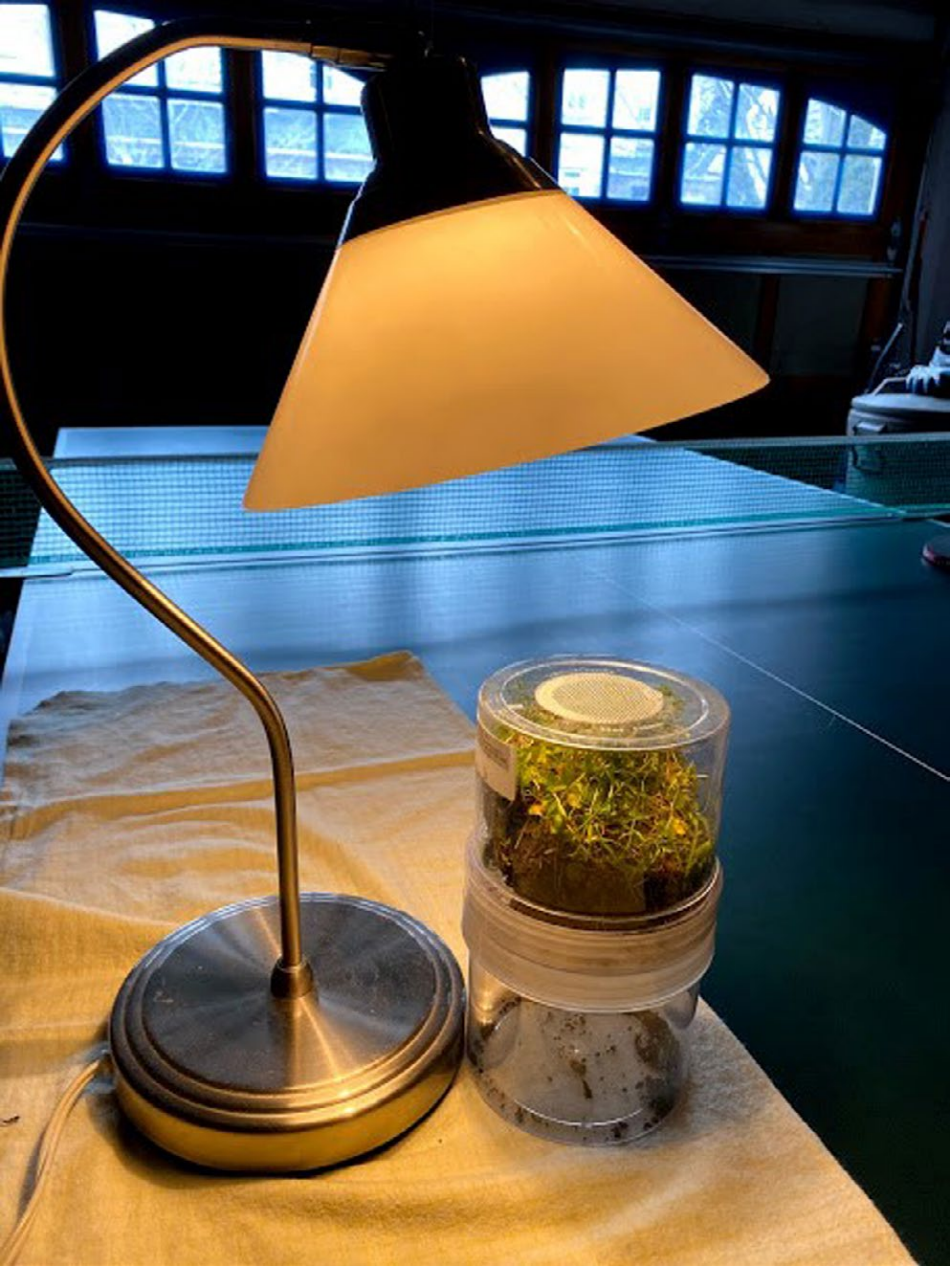
An example Burlese funnel setup in a student's garage. Photograph credit: M. Jurj

**Figure 5:**
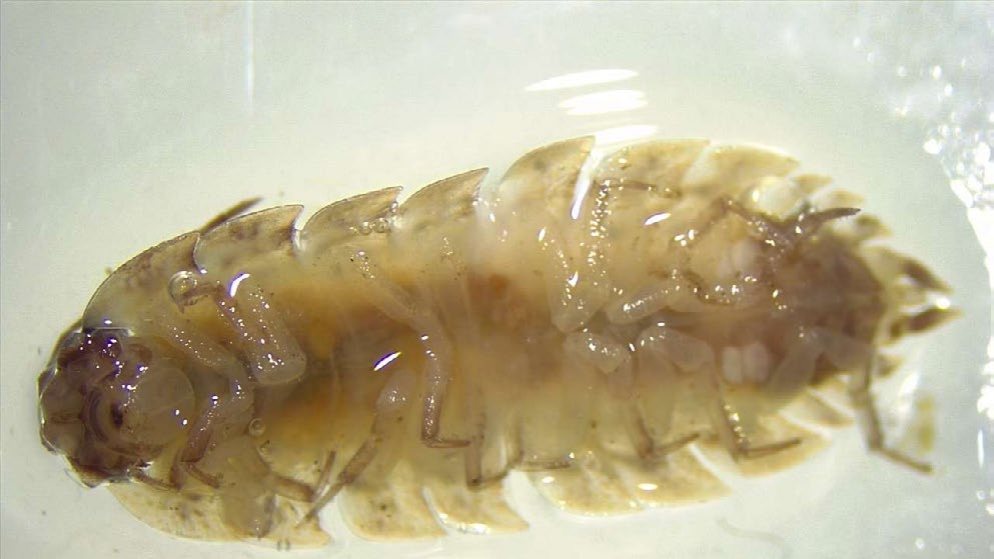
An image of one Isopod specimen collected, identified, and photographed by a student using the dissecting scope. Photograph credit: M. Jurj

Other than the identification of insects, this taxon is easier for students to monitor in their own backyards as many of the collection protocols are passive, and even in an urban environment, a good sample size is almost guaranteed. For these field exercises, students were provided with a sweep net, 2 simple pitfall traps, a Berlese funnel, strainers and whirlpaks for collection, a dissecting scope and several online keys to the Orders for identification. I tried to use the interactive videoconferencing as much as possible, for example, to show students the easiest way to dig a pitfall trap without destroying the surrounding habitat and how to best transfer samples from the pitfall into a whirlpak. Following several short online instructional sessions, students conducted the following field exercises; one sampling session of sweep netting, 4 consecutive days of pitfall trapping with daily collections (Figure 3) and one turf sample collection to test the Burlese funnel (Figure 4). As the identification of insects was to Order only, the exercise was independent, however, the dissecting scopes had built in cameras (EZ4 W, Leica, Heerbrugg, Switzerland) therefore students were able to share pictures of their specimens to confirm identifications within the group (Figure 5). Students collected and identified 202 individuals from a total of 11 Orders across all yards.

## 6 URBAN MAMMALS

**Figure 6:**
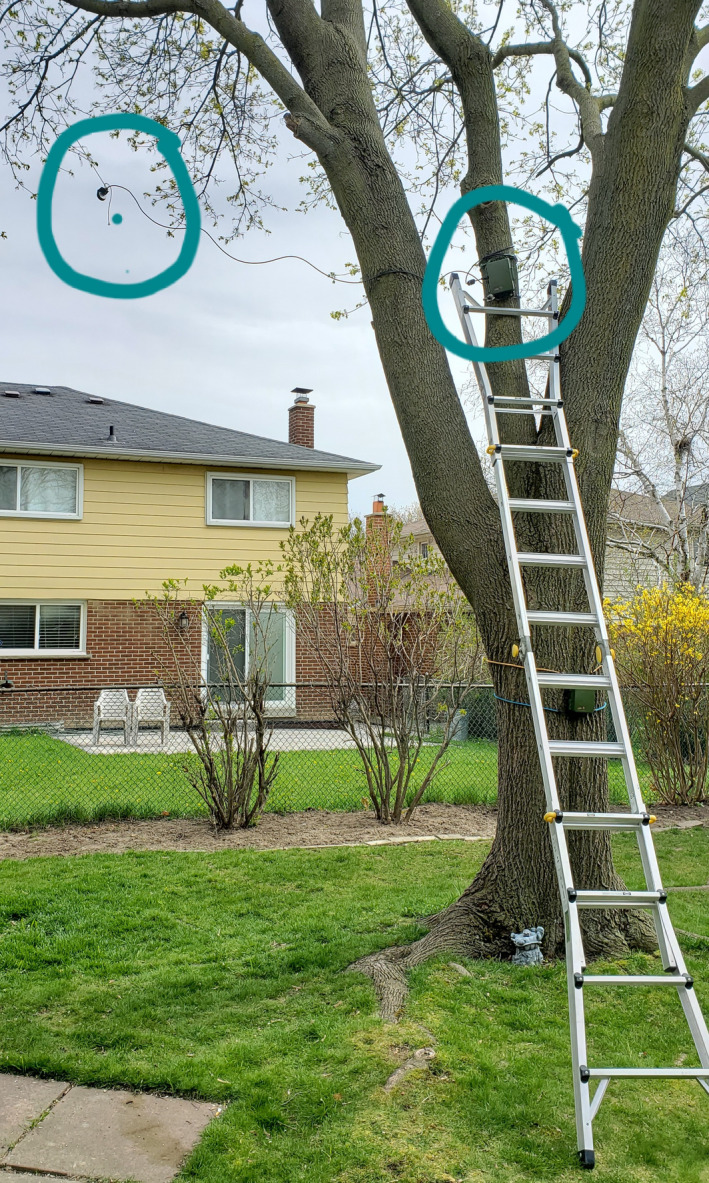
An example of the bat bioacoustics recorder deployment in one students’ backyard. The recorder is attached to the tree while the ultrasonic microphone is hanging from one of the branches. Photograph credit: S. Nichols

To survey mammal activity, students deployed camera traps (Moultrie M‐Series Digital Game Camera, Moultrie Products, Calera, AL, USA)) over a period of 48 hr in their yards. To avoid privacy concerns, cameras were set in an area where they would only be capturing images on the private property, not of the private neighbors or the general public passing by. For example, students were not permitted to post a camera on their fence facing outside of their yard. Unfortunately, due to some technical issues (*SD* cards provided that were not well suited to the cameras), only four students were able to capture images of wildlife on their cameras. Of those that did work, a minimum of four species were noted. This logistical challenge, however, was a great example for students of what often happens in the real world (see Meek et al., 2015). Students also deployed ultrasonic bioacoustics recorders to detect bats in their yards. The recorders (Song Meter SM4‐BAT Ultrasonic Recorder, Wildlife Acoustics, Maynard, WI, USA) were set to record from sunset to sunrise everyday for 4 days (Figure 6). Students previewed a sample of the data from the bat recorders using the Kaleidoscope 5.1.9i (Wildlife Acoustics Inc) but they were not analyzed further due to time constraints.

## 7 URBAN HABITAT

Students were required to provide detailed descriptions of the habitat surveyed throughout the course at multiple scales. Each student was provided with a Diameter at Breast height (DBH) tape, a compass with declination (for estimating tree height), a tree guide, and several online resources and encouraged to use apps such as iNaturalist to aid in identification. Each student provided a basic description of their site including latitude and longitude, size in area (meters squared), and cardinal direction of the yard (south facing, north facing etc). For the small‐scale habitat, survey students were asked to survey all the trees in their yards, identify them to species, estimate tree height and measure DBH 1.3 m from the ground. Students were also required to survey shrubs, identify them to species and measure shrub height. An estimate of percentage ground cover was also estimated for tree canopy, shrubs and total natural space to have an idea of total greenspace available. Finally, for a large‐scale habitat survey, students used Google earth (earth.google.com/web/) to estimate greenspace with a 500 m × 500 m quadrat centered on their site. Again, I took advantage of the interactive videoconferencing as much as possible, for example, to show students how to use a compass, and how to use the declination to estimate tree height using the compass and the Pythagorean theorem.

## 8 DATA SHARING AND GROUP PROJECTS

All data collected during the above‐mentioned field exercises were entered into an online shared folder. Each student was responsible for entering their own data and all data were available for the group research projects. Students were also encouraged to share photographs.

Students paired up for the group research projects which were broken down into 4 stages: (a) individual: data collection in their own backyards described above, data entered into shared google sheets file, (b) groupwork: write a research proposal (introduction and methods) based on data collected, (c) groupwork: write the outline of a research paper incorporating my comments on the proposal, and adding a new results section, (d) individual: incorporate my comments on outline and provide a final individual research paper—introduction, methods, results, discussion.

Given that the group research project accounted for 80% of the students’ grade (proposal, presentation, first draft, and final draft combined), discussions regarding potential research topics began on the first day of the course. In general, guidelines regarding topics were very open. The sampling techniques explored during the course estimated presence/absence, abundance or diversity of plants, mammals, insects, and birds in an urban environment. As we explored new sampling techniques each day, I encouraged students (during our videoconferencing sessions) to draw upon their previous coursework in ecology and think about the fundamental ecological theories that could be tested using the type of data we were collecting. Several students recalled the theory of Island Biogeography and quickly understood that they had the potential to test some aspects of this theory in an urban environment by investigating the effects of greenspace availability (or patch size) on species richness (Appendix S1). Other students were more interested in effects of environmental stressors such as noise pollution or weather variables on bird behavior (song; Appendix S1). Despite being limited to their own backyards, the ability to collect data as a group at 8 different sites provided ample opportunity for research projects with a strong foundation in ecological theory. In previous courses, when students were conducting their research on campus, they had the additional task of uncovering the natural small‐scale variation in habitat or environmental stressors on campus, hence the research topics tended to be more variable, but equally impressive (Appendix S1).

## 9 INCREASING CLASS SIZE

Though I had the pleasure of working with a very small group of students for this particular online transition, the course design could be easily scaled up to almost any class size with a few minor modifications. The equipment boxes and pickup thereof would likely not work for larger class sizes. For starters, the equipment was very expensive, and the pickup and drop‐off of boxes required a good deal of preparation and time for both students and staff (especially during a pandemic). For a larger class, the required equipment would need to be simplified, and perhaps listed as required course materials for students to buy, analogous to the textbooks, dissection kits and lab coats they normally need to buy for a course with laboratory sections. Key items to list as required course materials, that students would be likely to use again in other ecology courses, would be (a) binoculars, (b) a compass, and (c) a magnifying lens. Other items used in the course, such as pitfall traps, sweep nets and Burlese funnels, can be made from household items, and some activities (camera trapping and bioacoustics monitors) could be replaced by low‐cost alternatives (see Appendix S2 for a list of ideas). Though it would be possible to make the field exercises work at a larger class size, I would still recommend keeping the class size at a maximum of 20 students for an online field course like this. My group was small enough that it was still possible to foster a feeling of comradery, even though we were online. At a much larger group size, when you cannot see everyone on the screen at the same time, this connection would most likely be lost.

## 10 CONCLUSION

In the context of COVID‐19, where students had been isolated at home for almost 2 months, it was clear that students were eager and excited to have something different to do at home. The feeling of excitement was evident in the first videoconference meetings. Students enjoyed the casual time at the beginning of the online sessions to catch up with each other and were eager to share their new observations each morning. The course was certainly successful in this manner. Though as interactive as possible, the online field course may never be able to achieve the same social benefits of a live field course, however, the educational experience, if well planned, can certainly compare. All students were introduced to several new field skills, collected data independently, and worked together to compile these data and conduct interesting research projects. In conclusion, an online field course that incorporates direct experience with the natural environment is possible and should no long be considered an oxymoron.

## CONFLICT OF INTERESTS

There are no competing interests to document.

## AUTHOR CONTRIBUTION

**Laura McKinnon:** Conceptualization (lead); Investigation (lead); Methodology (lead); Writing‐original draft (lead); Writing‐review & editing (lead).

## Supporting information


**Appendix S1.**
**Appendix S2.**

## Data Availability

Not applicable.
