# Supplementary material for: YIMBY—Yes, In My BackYard!—The successful transition to a local online ecology field course
Source: Ecol Evol. 2020 Oct 7;10(22):12542–8. doi: 10.1002/ece3.6881 (PMC7679549; doi:10.1002/ece3.6881)
Supplement: Supplementary file 1 — Appendix S1. Appendix S2. [file ECE3-10-12542-s001.docx]

APPENDIX 1. DESCRIPTION OF RESEARCH PROJECT TOPICS PAST AND PRESENT.

**RESEARCH PROJECT TOPICS DURING THE ONLINE FIELD COURSE (2020)**

- Occurrence rates of American Robin (*Turdus migratorius*) Song in Relation to Varying Weather Patterns

Students estimated call rates of American Robins (calls/hour) at sunrise and sunset using bioacoustics recorders deployed in the 8 backyard sites over the 10-day period of data collection (308, 1-hour wav files!). They then compared the song rates between the 5 main weather conditions experienced during the study period.

- Effects of noise pollution on song frequency of American Robins (*Turdis migratorius*)

Students estimated the call rates for American Robin songs (calls/hour), the minimum and maximum frequency of American Robin calls (in Hz), and background noise levels at each site (in dB), using the same bioacoustics data mentioned above. This group also estimated the distance to the nearest road for each site. They then tested for 1) an effect of distance to the nearest road on call rates, and 2) an effect of background noise levels on the minimum and maximum call frequency (Hz) of American Robins.

- Bird species richness across a gradient of greenspace availability in residential areas

Students estimated species richness based on point count surveys conducted at sunrise and sunset within the 8 backyard sites over an 8-day period of data collection. This group also initiated incidental bird surveys at varying times of the day across the sites during the same period. They then used the estimates of greenspace availability measured for each site (part of the habitat exercise), to test for an effect of the greenspace availability on bird species richness.

- The Effect of Available Greenspace on Avian Biodiversity

Students estimated species richness based on a combination of the point count surveys and bioacoustic data mentioned above. They then used the estimates of greenspace availability measured for each site (part of the habitat exercise), to test for an effect of the greenspace availability on bird species richness.

**PREVIOUS RESEARCH PROJECT TOPICS BY SUBJECT (2017, 2018, 2019).**

Animal Behaviour

- The effects of recorded alarm calls of Cooper’s Hawk (*Accipiter cooperii*) on the foraging behaviour of the American Robin (*Turdus migratorius*)
- Effects of Heterospecific Alarm Calls on the Behaviour of American Robins and Northern Cardinals
- The effect of anthropogenic noise on anti-predator behaviour of the southern Ontario Eastern gray squirrel (*Sciurus carolinensis*)
- L’effet de l’odeur sur l’efficacité de trappage des insectes

Predator-prey interactions

- The Population Density of American Robins (*Turdus migratorius*) in Relation to Earthworm (*Lumbricus terrestris*) Availability
- Nest-site Selection in Killdeer (*Charadrius vociferus*): Evaluating the Role of Insect Availability
- L’effet du pH du sol sur le nombre des lombrics (*Lumbricidae*) et les habitudes alimentaires subséquents des merles d'Amérique (*Turdus migratorius*)

Urban Biodiversity

- Effects of urbanization on soil fauna biodiversity
- Effects of habitat type and environmental conditions on species distribution of pollinating flying insects.
- The Influence of Edge Effects on Insect biodiversity
- Effects of Soil Properties on Insect Diversity and Richness
- Impacts of the invasive shrub *Rhamnus cathartica* on urban woodlot plant diversity
- Les effets des différentes conditions environnementales sur la biodiversité et l’abondance des insectes en région urbanisée.

APPENDIX 2: List of suggested online field course materials by class size.

|  | SMALL (<20)  ONLINE FIELD COURSE | LARGE (>20)  ONLINE FIELD COURSE | FREE ONLINE APPLICATIONS |
| --- | --- | --- | --- |
| BIRDS | Binoculars provided | Students purchase binoculars (minimum estimated cost $50) |  |
|  | Students encouraged to use free online bird and bird song identification applications | | Merlin Bird ID (https://merlin.allaboutbirds.org/), Raptor ID (https://hawkwatch.org/blog/item/1164-raptor-id-app-now-free), Audubon Bird Guide (https://www.audubon.org/app), iNaturalist (https://www.inaturalist.org/),  SongSleuth (https://www.songsleuth.com/#/) |
|  | Bird field guide provided |  |  |
|  | Provided Bioacoustic monitors |  |  |
| INSECTS | Pitfall traps, sweep net and Burlese funnel provided. | Students use household items to make pitfall traps, sweep nets and Burlese funnels. |  |
|  | Dissecting scope provided | Students purchase magnifying lens (minimum estimated cost price $20) |  |
|  | Encouraged use of free online insect identification applications. | | iNaturalist (https://www.inaturalist.org/),  Insect Picture  (https://play.google.com/store/apps/details?id=com.glority.pictureinsect&hl=fr) |

|  | SMALL (<20)  ONLINE FIELD COURSE | LARGE (>20)  ONLINE FIELD COURSE | FREE ONLINE APPLICATIONS |
| --- | --- | --- | --- |
| AMPHIBIANS | Encouraged use of free online amphibian call ID applications | | Frog Calls (https://play.google.com/store/apps/details?id=com.johneyboy.frogcalls&hl) |
| MAMMALS | Camera traps provided. | Students conduct scat surveys and/or other surveys using signs of of mammal activity (browse surveys, prints etc). |  |
| HABITAT | Clinometer (to measure tree height) provided | Students purchase a compass with clinometer  (minimum cost $50) |  |
|  | DBH (diameter at breast height) tapes and 50m measuring tape provided | Student purchase measuring tape (minimum cost $10), to be used to measure transects, quadrats and estimate DBH |  |
|  | Encouraged use of online plant identification applications | | PlantNet (<https://plantnet.org/>),  PlantSnap (<https://www.plantsnap.com/>),  iNaturalist (https://www.inaturalist.org/) |
|  | Tree and shrub field guides provided |  |  |
